# Lapatinib monotherapy in patients with relapsed, advanced, or metastatic breast cancer: efficacy, safety, and biomarker results from Japanese patients phase II studies

**DOI:** 10.1038/sj.bjc.6605343

**Published:** 2009-10-20

**Authors:** M Toi, H Iwata, Y Fujiwara, Y Ito, S Nakamura, Y Tokuda, T Taguchi, Y Rai, K Aogi, T Arai, J Watanabe, T Wakamatsu, K Katsura, C E Ellis, R C Gagnon, K E Allen, Y Sasaki, S Takashima

**Affiliations:** 1 Department of Surgery, Graduate School of Medicine, Kyoto University, 54 Kawaracho, Shogoin, Sakyo-ku, Kyoto 606-8507, Japan; 2 Department of Surgery, Tokyo Metropolitan Komagome Hospital, 3-18-22 Honkomagome, Bunkyo-ku, Tokyo 113-8677, Japan; 3 Department of Breast Surgery, Aichi Cancer Center Hospital, 1-1 Kanokoden, Chikusa-ku, Nagoya, Aichi 464-8681, Japan; 4 Breast Oncology Internal Medicine, National Cancer Center Hospital, Tsukiji 5-1-1, Chuo-ku, Tokyo 104-0045, Japan; 5 Department of Surgery, The Cancer Institute Hospital of JFCR, Ariake 3-10-6, Koto-ku, Tokyo 135-8550, Japan; 6 Department of Breast Surgery, St Luke's International Hospital, Akashi-cho 9-1, Chuo-ku, Tokyo 104-8560, Japan; 7 Breast Endocrine Surgery, Tokai University Hospital, 143 Shimokasuya, Isehara, Kanagawa 259-1193, Japan; 8 Breast Endocrine Surgery, Osaka University Hospital, Yamada-oka 2-15, Suita City, Osaka 565-0871, Japan; 9 Surgery, Sagara Hospital, 3-31 Matsubara-cho, Kagoshima, Kagoshima 892-0833, Japan; 10 Department of Surgery, Shikoku Cancer Center, 160 Kou, Minamiumemoto-cho, Matsuyama, Ehime 791-0288, Japan; 11 Medical Oncology, Tochigi Cancer Center, 4-9-13 Yonan, Utsunomiya, Tochigi 320-0834, Japan; 12 Female Internal Medicine, Shizuoka Cancer Center, 1007 Shimonagakubo, Nagaizumi, Shizuoka 411-8777, Japan; 13 Clinical Research, GlaxoSmithKline KK, 4-6-15, Sendagaya, Shibuya-ku 151-8566, Japan; 14 Clinical Oncology, GlaxoSmithKline, 1250 South Collegeville Road, Collegeville, PA 19426, USA; 15 Medical oncology, Saitama Medical University, 1397-1 Yamane, Hidaka, Saitama 350-1298, Japan

**Keywords:** lapatinib, monotherapy, HER2, metastatic breast cancer, biomarker, trastuzumab

## Abstract

**Background:**

HER2-positive metastatic breast cancer (MBC) relapsing after trastuzumab-based therapy may require continued HER2 receptor inhibition to control the disease and preserve the patients' quality-of-life. Efficacy and safety of lapatinib monotherapy was evaluated in Japanese breast cancer patients after trastuzumab-based therapies.

**Methods:**

In studies, EGF100642 and EGF104911 evaluated the efficacy and safety of oral lapatinib given 1500 mg once daily in patients with advanced or MBC. All patients progressed on anthracyclines and taxanes; HER2-positive patients had also progressed on trastuzumab.

**Results:**

For HER2-positive tumours (*n*=100), objective response rate was 19.0% (95% confidence interval (CI): 11.8–28.1) and clinical benefit rate (CBR) was 25.0% (95% CI: 16.9–34.7). One out of 22 HER2-negative tumour was documented as complete response (*n*=22). The median time-to-progression (TTP) in the HER2-positive and HER2-negative groups was 13.0 and 8.0 weeks (*P*=0.007); median overall survival was 58.3 and 40.0 weeks, respectively. The most frequent adverse event was diarrhoea. TTP and CBR were significantly associated with HER2 expression. Patients with tumours harbouring an H1047R PIK3CA mutation or low expression of PTEN derived clinical benefit from lapatinib.

**Conclusion:**

Lapatinib monotherapy had shown anti-tumour activity in Japanese patients with HER2-positive MBC that relapsed after trastuzumab-based therapy, including those with brain metastases. Patients benefiting from lapatinib may have biomarker profiles differing from that reported for trastuzumab.

Approximately, 30% of invasive breast cancers (BC) overexpress HER2, an adverse prognostic factor associated with aggressive histopathological parameters and worse clinical outcome (Slamon *et al*, 1987).

Despite the clinical breakthrough trastuzumab brought to the treatment of HER2-overexpressing BC, the majority of metastatic BC (MBC) does not respond to trastuzumab monotherapy, and controversy remains over the optimal therapeutic strategy after disease progression (Cobleigh *et al*, 1999; Vogel *et al*, 2002; Montemurro *et al*, 2006). Intrinsic and acquired trastuzumab resistance remains a concern (Nahta and Esteva, 2006; Xia *et al*, 2007). Additional concerns include increased incidence of brain metastasis and risk of both short- and long-term cardiac toxicity associated with trastuzumab therapy (Guarneri *et al*, 2006; Suter *et al*, 2007).

Lapatinib, an orally active, small molecule, reversible inhibitor of EGFR and HER2, is being evaluated for treatment of cancers with EGFR expression and HER2 overexpression, including cancers that are refractory to standard therapy. The simultaneous blockade of both receptors by lapatinib may be more effective than single receptor inhibition because of the propensity for HER family hetero-dimerisation and resultant signalling diversity (Mukherjee *et al*, 2007). Lapatinib, as monotherapy or in combination with chemotherapy, has promising anti-tumour activity in HER2 positive locally advanced or MBC as evidenced by efficacy outcome reported earlier. Japan and many other countries showed that lapatinib has efficacy in patients having HER2-positive BC with brain metastases (Xia *et al*, 2002). From 44 clinical trials, safety analysis indicated favourable cardiac toxicity profile of lapatinib with a low frequency of asymptomatic events that are reversible (Perez *et al*, 2008).

In Japan, two Phase II studies were conducted to evaluate the efficacy and safety of lapatinib monotherapy in advanced or MBC that had progressed on prior anti-tumour therapies, including trastuzumab.

## Materials and methods

### Study design

EGF100642 and EGF104911 were multicentre, single-arm, open-label studies involving 23 centres in Japan. Studies were designed in accordance with the Japanese Ministry of Health, Labor and Welfare Ordinance on Good Clinical Practice, to evaluate the efficacy and safety of lapatinib in patients with advanced or MBC. Studies were approved by the institutional review board of each participating institution. All patients provided written informed consent.

The primary endpoint was objective response rate (ORR: confirmed complete response (CR) and partial response (PR)) according to response evaluation criteria in solid tumours (RECIST) and determined by an independent review committee. Secondary efficacy endpoints included clinical benefit rate (CBR: CR, PR and stable disease (SD) for ⩾24 weeks); time-to-progression (TTP); and overall survival (OS). In EGF100642, patients were grouped, according to intra-tumoural HER2 expression, into cohort A or B (HER2 positive and HER2 negative, respectively). EGF104911 included patients with HER2-overexpressing tumours only. The planned accrual for EGF100642 and EGF104911 was 100 patients (40 in cohort A; 60 in cohort B) and 52 patients, respectively. Integrative analyses were conducted on efficacy, safety and biomarker data from the two studies.

### Patient eligibility

Eligible patients were female aged 20–74 years with confirmed advanced (stage IIIb or IIIc with T4 lesion) or MBC that had progressed on prior anthracycline- and taxane-containing regimens. All patients with HER2 overexpression had received ⩾6 weeks of prior trastuzumab. Eligible patients had measurable lesions as defined by RECIST; an Eastern Cooperative Oncology Group (ECOG) performance status of 0–2; left ventricular ejection fraction (LVEF) within the institutional normal range (or ⩾50%); adequate renal, hepatic and haematologic functions.

### Treatment schedule

Lapatinib 1500 mg was taken orally once daily in fasted conditions. Patients were treated until disease progression, unacceptable toxicity or consent withdrawal.

### Efficacy and safety assessments

Safety and efficacy assessments (including echocardiogram for LVEF) were performed at 4- and 8-week intervals, respectively, at treatment completion and 28 days after the last dose. All patients were followed for survival every 12 weeks until death. Adverse events (AEs) were assessed according to the National Cancer Institute Common Terminology Criteria for AEs version 3.0 (NCI CTCAE v3.0).

### Immunohistochemistry and fluorescent *in situ* hybridisation

Formalin-fixed, paraffin-embedded (FFPE) archived tumour tissue blocks (or sections) were required for enrolment. Intra-tumoural protein expression levels were determined by immunohistochemistry (IHC) and reported as an *H*-score (McCarty *et al*, 1986) excluding EGFR and HER2, which were reported by the assay defined ordinal score (Dako, Carpinteria, CA, USA). The following antibodies were used in the semi-quantitative IHC assays performed at Quest Diagnostics (Van Nuys, CA, USA): HER2 (HercepTest, Dako); EGFR (PharmDx, Dako) and at Pathway Diagnostics (Malibu, CA, USA): ErbB3 (NeoMarkers RB-9211, Thermo Fisher, Fremont, CA, USA); ErbB4 (#18-7329, Zymed, South San Francisco, CA, USA); IGF1R (NeoMarkers MS-641, Thermo Fisher); BCL2 (Dako). PTEN (Cell Signaling Technology, Danvers, MA, USA, #9559) analysis was performed at European Institute of Oncology (Milan, Italy). Deoxyribonucleic acid (DNA) fragmentation (TUNEL) was measured at Pathway Diagnostics using the ApopTag *in situ* assay (Chemicon International, Temecula, CA, USA). Oestrogen (ER) and progesterone (PgR) receptor results were from local laboratories and reported as positive or negative. HER2 gene amplification was determined by fluorescence *in situ* hybridisation (FISH) using the PathVysion kit (Vysis, Downers Grove, IL, USA) at Quest Diagnostics.

### Mutation assessments

DNA was extracted from FFPE tumour tissue; seven of the exons comprising the kinase domain of PIK3CA (exons 14–20) and exon 9 (helical domain) were amplified by polymerase chain reaction and sequenced using the ABI3730XL sequencer (Applied Biosystems, Foster City, CA, USA); somatic single nucleotide polymorphisms (SNPs) were identified by sequence analysis and mapping the results to the PIK3CA GenBank reference sequence NM_006218.

### HER2 extracellular domain

Serial blood samples were collected at baseline, every 4 weeks, and at study conclusion. Serum HER2 extracellular domain (ECD) levels were analysed at Quest Diagnostics using an HER-2/neu Microtiter enzyme-linked immunosorbent assay kit from Oncogene Science (Cambridge, MA, USA).

### Statistical design and analysis

The hypotheses for both studies were based on expected ORR (⩾15%), accordingly the sample sizes (calculation using SAS version 8.2, UNIX/Solaris) ensured ⩾74% power to deem lapatinib monotherapy effective by correctly rejecting the null hypothesis (H0: *P*<0.05) and accepting the alternative hypothesis (H1: *P*>0.15). The efficacy endpoints were estimated using two-sided 95% confidence interval (CI); TTP and OS were summarised using the Kaplan–Meier method.

Biomarkers were related to TTP using Cox proportional hazards models, and to clinical response using logistic regression. Correlations between biomarkers were evaluated using Spearman's rank correlation coefficient. Statistical tests were considered significant if *P*<0.05. Analyses were conducted using SAS v9.1.3.

## Results

### Patients

Between June 2004 and October 2006, 122 patients (64 and 58 from EGF100642 and EGF104911, respectively) were enroled; 100 had HER2-positive tumours (defined as IHC3+ or IHC2+ and FISH positive) and 22 (cohort B) had HER2-negative tumours (defined as IHC 0/1+ or FISH negative). Although the planned patient number for cohort B was 60, accrual stopped based on minimal efficacy of lapatinib monotherapy reported in HER2-negative BC (Burstein *et al*, 2008). Out of 122 patients, 119 discontinued therapy; 108 (91%) because of disease progression, 8 (7%) because of AEs and 3 (3%) withdrew consent. Three patients with HER2-positive tumours remained on study treatment.

Patient baseline characteristics are shown in Table 1. Over 80% had at least three prior anti-tumour regimens. All patients received prior anthracyclines and taxanes. All but 2% of HER2-positive patients had previously received trastuzumab for advanced or metastatic disease. The majority (75%) had received trastuzumab within 8 weeks before enrolment.

The median duration of treatment was 13.9 weeks in patients with HER2-positive tumours and 7.4 weeks in those with HER2-negative tumours.

### Efficacy

In HER2-positive patients, 1 CR (1%) and 18 PRs (18.0%) were documented, for an ORR of 19.0% (95% CI: 11.8–28.1); SD and progressive disease (PD) were observed in 38% (38 out of 100) and 41% (41 out of 100), respectively; two patients had an unknown response. In patients with HER2-negative tumours only one patient responded (CR; ORR=4.5%), the majority (68%) had PD and 14% had short-term SD. CBR in HER2-positive patients was 25.0% (95% CI: 16.9–34.7) and 4.5% in HER2-negative patients. The median TTP was 13.0 weeks (25th, 75th percentiles: 7.8, 24.1) in HER2-positive patients and 8.0 weeks (25th, 75th percentiles: 5.4, 8.4) in HER2-negative patients. The median OS was 58.3 weeks (25th, 75th percentiles: 26.2, 64.8) in HER2-positive patients and 40.0 weeks (25th, 75th percentiles: 18.3, 69.3) in HER2-negative patients.

In the HER2-positive group, 10 patients had brain metastases at baseline, 8 had no brain lesion progression of these 2 had ORR of PR, whereas brain lesions progressed in 2 patients.

### Safety

Among 122 patients evaluable for safety, 120 (98.4%) experienced at least 1 AE, most were Grade 1 or 2. The Grade 4 AEs (increased bilirubin and *γ*-glutamyl transferase) occurred in a patient with concurrent progression of liver metastases. The AEs with ⩾20% incidence are presented in Table 2.

There were 27 non-fatal serious AEs in 19 patients, 13 were possibly related to lapatinib. The only lapatinib-related serious AEs occurred in more than one patient were anorexia (*n*=3, 2.5%) and left ventricular dysfunction (*n*=2, 1.6%). Both patients experiencing left ventricular dysfunction remained on lapatinib and the event later resolved.

Three patients died because of disease progression. One patient experienced fatal AEs (increase in *γ*-glutamyl transferase and bilirubin); the events, deemed unrelated to lapatinib, were due to liver metastases progression. The other two experienced ‘unexpectedly rapid’ disease progression; one in a HER2-negative patient.

### Biomarkers

The following biomarkers had results available in 95–100% of tumour samples: EGFR; HER2 (Table 3); ER; PgR. ErbB3, PTEN, IGF1R and BCL2 were well represented for EGF100642 (>80%), but were sparse for EGF104911 (⩽40%). The remaining biomarkers: Heregulin, TUNEL and Survivin, were represented in 55% or fewer samples, with particular scarcity in EGF104911 (<20%).

**Figure 1:**
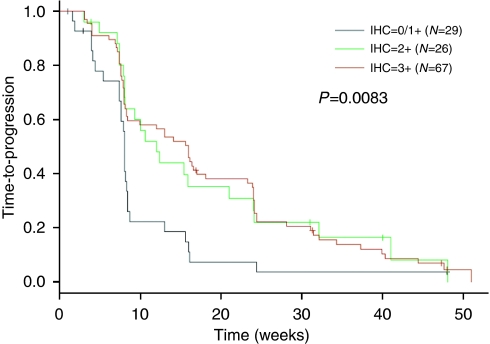
Kaplan–Meier estimates for TTP in the combined studies. TTP is stratified by HER2 IHC Score. Median IHC 0/1=8 weeks; IHC 2+=12 weeks; IHC 3+=16 weeks. (log rank *P*=0.0083); 23 out of 26 HER2 IHC2+ tumours were FISH positive, 3 out of 26 tumours were non-evaluable by FISH.

The HER2 IHC score was strongly associated with TTP (*P*=0.0083, Figure 1). TTP increased monotonically with increasing HER2 IHC score. CBR was higher in HER2 IHC 2+ and 3+ tumours (*P*=0.0168). The proportion of patients deriving clinical benefit from lapatinib in IHC 0, 1+, 2+ and 3+ groups was 4% (1 patient), 0%, 23%, and 73%, respectively. In 23% of patients experiencing clinical benefit from lapatinib and with IHC2+ tumours, all were HER2 FISH positive.

**Figure 2:**
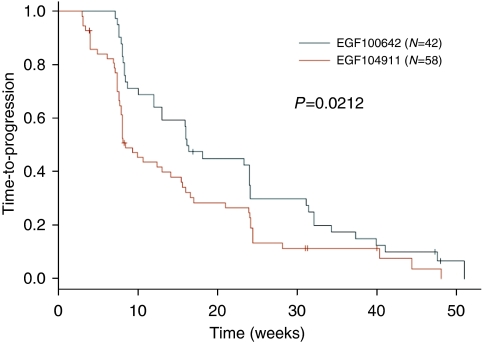
Kaplan–Meier estimates for TTP. TTP is significantly longer in EGF100642 HER2-positive patients *vs* EGF104911 patients (median TTP for EGF100642=16.3 weeks; EGF104911=8.4 weeks; *P*=0.0212).

TTP and CBR differed between the studies. HER2-positive patients in EGF100642 experienced longer TTP compared with EGF104911 (*P*=0.0212, Figure 2). Cohort A (EGF100642) had a higher CBR than EGF104911 (*P*=0.0385, Table 4). The efficacy differences between the studies may be explained by the difference in the frequency of ER and PgR expression. In EGF100642, 17% of HER2-positive tumours were ER positive, whereas 43% were ER positive in EGF104911; PgR positive was detected in 15% and 35%, respectively. Although CBR was not significantly associated with hormone receptor status (*P*=0.100), PgR status had a significant effect on TTP (*P*=0.016), whereas ER status did not (*P*=0.3188). Patients with PgR-positive tumours regardless of ER status (*n*=26) had the shortest median TTP (8.4 weeks), whereas patients with PgR-negative tumours had similar median TTP (15.4 weeks for ERnegative, PgR negative, *n*=60; 17 weeks for ER positive, PgR negative, *n*=12).

**Figure 3:**
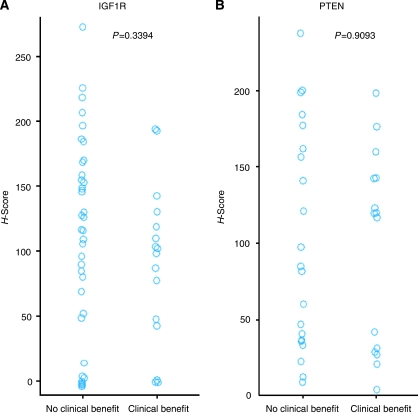
Distribution of *H*-scores as measured by IHC, grouped according to patient response (clinical benefit=CR; PR; s.d.⩾24 weeks; no clinical benefit=s.d. <24 weeks; PD; NE). (**A**) IGF1R protein expression levels (*n*=58; *P*=0.3394). (**B**) PTEN protein expression levels (*n*=36; *P*=0.9093).

EGFR and IGF1R expression were not significantly associated with efficacy parameters. IGF1R expression did not preclude patients from deriving benefit from lapatinib (*n*=58, *P*=0.3394, Figure 3A). PTEN expression did not associate with lapatinib response; in patients with HER2-positive tumours (EGF100642, *n*=36), CBR was not dependent on PTEN (*P*=0.9093), thus patients with tumours expressing low levels of PTEN derived benefit from lapatinib (Figure 3B).

For exploratory purposes, biomarkers were tested for correlation with each other in the HER2-positive patients. Significant correlations were found between BCL2 and IGF1R (*r*=0.33, *P*=0.015), ErbB3 and Heregulin (*r*=0.39, *P*=0.012) and IGF1R and PTEN (*r*=0.62, *P*=0.0003).

Biomarkers were compared with ER and PgR status in HER2-positive patients using the Wilcoxon rank sum test. BCL2 levels were significantly higher in ER-positive patients (*n*=52, *P*=0.017) and PgR-positive patients (*n*=51, *P*=0.025). Heregulin (*n*=40, *P*=0.005) and Survivin (*n*=22, *P*=0.047) were found to be higher in PgR-negative patients.

PIK3CA gene (exon 9; 14–20) mutation analysis was conducted in EGF100642; 30 tumour samples had sufficient quantities for of genomic DNA. Owing to the low quantities of genomic DNA in all tumour samples, exon 20 was the highest priority because of the presence of the well-characterised H1047R kinase-activating mutation located in this exon and detected in BC (Kang *et al*, 2005). Exon 20 mutation analysis was completed in 29 samples; in 3 tumours the H1047R mutation was detected. Lapatinib response in the three patients (HER2 positive) whose tumours harboured the H1047R mutation included one durable PR (32 weeks), and two SD (16 weeks). Mutation analysis on the remaining exons identified two SNPs in two different tumour samples resulting in non-conservative amino acid substitutions (exon 15: E767K; exon 18: T898I). Although both substitutions were predicted to be structurally tolerated and speculated to not directly affect PIK3CA kinase activity, the indirect functional implications were unknown. The patients whose tumours contained these SNPs derived clinical benefit from lapatinib: one (HER2 positive) SD for ⩾24 weeks; one (HER2 negative) CR for 23 weeks. In tumours with evaluable results (10 out of 30), no SNPs resulting in an amino acid change were detected in exon 9.

The distribution of baseline serum ECD (bECD) values showed that levels appear to be higher in patients with HER2-positive tumours (median: 44.5 ng ml^−1^; 25th, 75th percentiles: 15.2, 114.3) *vs* HER2-negative tumours (median: 9.9 ng ml^−1^; 25th, 75th percentiles: 8.0, 12.4); however, bECD did not predict response. After adjusting for HER2 status in the pooled sample, bECD (entered into the model as log of bECD) was not a significant predictor of TTP in a proportional hazards model (*P*=0.568). In a similar model, neither were bECD levels predictors of TTP within HER2-negative (*P*=0.531) or HER2-positive patients (*P*=0.582), nor were bECD levels predictors of TTP in the HER2-positive patients when adjusted for ER/PgR status (*P*=0.896).

## Discussion

Lapatinib monotherapy was efficacious in treating HER2-overexpressing advanced or MBC that relapsed after trastuzumab treatment. Lapatinib benefit was confined mostly to HER2-positive tumours (ORR was 19%; CBR was 25.0%) and confirmed previous reports (Geyer *et al*, 2006; Gomez *et al*, 2008). In this heavily pretreated population with refractory HER2-positive BC, treatment with single-agent lapatinib resulted in a median TTP of 13.0 weeks and median OS of 58.3 weeks. Median progression-free survival was identical to TTP (13.0 weeks for the HER2-positive population), as no time-to-event at the time of data cut-off occurred because of death.

TTP and CBR differed between the studies. Patient characteristics were similar in both studies except for differences observed in the distribution of patients with hormone-receptor-positive BC, although expression was not confirmed by central review. This small dataset suggests that lapatinib may affect TTP based on PgR status. PgR expression and activity, whereas dependence on classical ER genomic signalling has been shown to be regulated by growth factor signalling (Arpino *et al*, 2008). Additional reports have suggested that negative PgR expression indicates high growth factor activity (Cui *et al*, 2005). Perhaps in the context of HER2 overexpression and ER positivity, low or absent PgR expression may be a predictor of tumour dependence on HER2 activity and thus sensitivity to HER2 inhibition with agents such as lapatinib.

Lapatinib response may be independent of PTEN, PIK3CA and IGF1R, as supported by lapatinib responses observed in these studies and demonstrated in preclinical studies (Nahta *et al*, 2007; Xia *et al*, 2007). Patients with HER2-positive tumours having PIK3CA amino acid changes derived benefit from lapatinib. Additionally, patients with low PTEN, or expressing IGF1R also derived benefit from lapatinib. These findings, albeit in a small cohort, suggested that lapatinib anti-tumour activity may depend on a cellular milieu different from trastuzumab, which reportedly has decreased activity in tumours with the aforementioned characteristics (Nahta and Esteva, 2006). Ongoing clinical trials in early and advanced BC have been designed to elucidate lapatinib mechanism of action, identify predictive biomarkers and further define the differences in activity compared with other HER2 inhibitors.

In the HER2-negative cohort, one patient experienced a durable CR (23 weeks) and had a primary tumour with the following molecular characteristics: HER2 negative and EGFR negative; ER positive and PgR positive; low expression of PTEN and PIK3CA (E767K) SNP. Biomarker evaluations were performed on the primary tumour and not the recurrent site; hence, it is interesting to speculate that the genotype/phenotype of treated tumour was modified at recurrence, supporting the practicing biopsy on relapse.

Brain metastases is an area of significant unmet medical need, as up to one-third of HER2-overexpressing MBC patients develop central nervous system metastases after trastuzumab treatment (Bendell *et al*, 2003; Clayton *et al*, 2004). In this study, 10 patients had brain metastases at baseline and of these 8 experienced either systemic PR or SD, indicating that lapatinib monotherapy is efficacious in HER2-positive BC patients with brain metastases, thus supporting previous reports (Lin *et al*, 2008).

Trastuzumab treatment is associated with cardiac dysfunction, with up to 28% of patients experiencing such events (Guarneri *et al*, 2006), although asymptomatic LVEF declines were more common than symptomatic heart failure (Marty *et al*, 2003). In this study, three patients experienced LVEF decrease (2.4%); however, with mild severity (Grade 1). These data were similar to that observed from 44 lapatinib clinical trials, in which the incidence was reported as 1.6% (60 out of 3689 lapatinib-treated patients) (Perez *et al*, 2008).

The results in Japanese patients showed that lapatinib was well tolerated and an effective treatment for HER2-positive BC (ORR=19%; CBR=25%), despite disease progression on trastuzumab-based therapy. Although low ORR (4.3; 7.7%) were observed in similar patient populations who were mostly Caucasian (Burstein *et al*, 2008; Blackwell *et al*, 2009); however, neither intrinsic nor extrinsic factors were evaluated to determine the efficacy differences. In first-line patients with trastuzumab-naïve, HER2-positive advanced or MBC, lapatinib monotherapy resulted in 24% ORR (Gomez *et al*, 2008). Lapatinib combination therapy has promising efficacy as treatment for HER2-positive advanced or MBC, including treatment-naïve MBC (Geyer *et al*, 2006; Di Leo *et al*, 2008; O'Shaughnessy *et al*, 2008; Johnston *et al*, 2008a, 2008b). The outcome of this study complements the efficacy reported earlier for lapatinib and confirms that lapatinib offers a beneficial therapeutic option to patients with HER2-overexpressing BC, including those with brain metastases. The efficacy for the tumours having the altered PI3K pathway may provide a novel possibility in the treatment of HER2-positive BCs.

## Conflict of interest

The authors declare no conflict of interest.

## Figures and Tables

**Table 1:** Patient characteristics – demographics and disease history

**Characteristic**	**HER2 positive *n*=100**	**HER2 negative *n*=22**	**Total population *n*=122**
Median age, years	55.0	52.5	55.0
			
*ECOG PS, n (%)*			
0–1	97 (97)	19 (86)	116 (95)
2	3 (3)	3 (14)	6 (5)
			
*Disease stage, n (%)*			
IIIB/IIIC (T4)	1 (1)	2 (9)	3 (2)
IV	99 (99)	20 (91)	119 (98)
			
*Prior anti-tumour therapy, n (%)*			
Anthracyclines	100 (100)	22 (100)	122 (100)
Taxanes	100 (100)	22 (100)	122 (100)
Trastuzumab	100 (100)	0	100 (82)
Neoadjuvant^a^	2 (2)	0	2 (2)
Adjuvant^b^	4 (4)	0	4 (3)
Advanced/metastatic	98 (98)	0	98 (80)
			
*No. of metastatic sites, n (%)*			
⩾3	43 (43)	9 (41)	52 (43)
2	36 (36)	6 (27)	42 (34)
1	21 (21)	7 (32)	28 (23)
			
*Hormone receptor status, n (%)*			
ER+ and/or PgR+	38 (38)	14 (64)	52 (43)
ER− and PgR−	60 (60)	8 (36)	68 (56)
Unknown (ER and/or PgR)	2 (2)	0	2 (2)

Abbreviations: ECOG PS=European Cooperative Oncology Group Performance Status; ER=oestrogen; PgR=progesterone.

a Patients received trastuzumab for neoadjuvant and advanced/metastatic setting.

b Two patients received trastuzumab for adjuvant and advanced/metastatic disease, two patients received in adjuvant setting only.

**Table 2:** Incidence of common adverse events reported in ⩾20% of patients by maximum NCI CTCAE toxicity grade

	**All grades**		**Grades 3/4**	
**MedDRA preferred term**	**No patients**	**%**	**Number of patients**	**%**
Any event	120	98.4	41	33.6
Diarrhoea	86	70.5	7	5.7
Rash	59	48.4	1	0.8
Stomatitis	54	44.3	0	0
Nausea	48	39.3	1	0.8
Anorexia	48	39.3	10	8.2
Fatigue	47	38.5	4	3.3
Pruritus	36	29.5	0	0
Nasopharyngitis	33	27.0	0	0
Aspartate aminotransferase increased	28	23.0	5	4.1
Dry skin	27	22.1	0	0
Vomiting	27	22.1	2	1.6
Pyrexia	24	19.7	1	0.8
Weight decreased	22	18.0	0	0
Alanine aminotransferase increased	21	17.2	3	2.5
Blood alkaline phosphatase increased	20	16.4	4	3.3
Dyspnoea	20	16.4	2	1.6

Abbreviations: MedDRA=Medical Dictionary for Regulatory Activities; NCI CTCAE=National Cancer Institute Common Terminology for Adverse Events.

**Table 3:** Distribution of EGFR and HER2 biomarker IHC scores

	**EGF100642**		**EGF104911**
	**HER2 positive^a^ (*n*=42)**	**HER2 negative (*n*=22)**	**HER2 positive^a^ (*n*=58)**
*HER2 IHC score, n (%)*			
0	0	16 (73)	2 (3)
1+	2 (5)	5 (23)	4 (7)
2+	6 (14)	1 (5)	19 (33)
3+	34 (81)	0 (0)	33 (57)
			
*FISH amplification, n (%)*			
Positive (⩾2)	39 (93)	1 (5)	53 (91)
Negative (<1.8)	0	7 (32)	1 (2)^b^
Non-evaluable	3 (7)^c^	13 (60)	3 (5)^b^
Borderline (1.8–<2)	0	1 (5)^d^	1 (2)^b^
			
*EGFR IHC score, n (%)*			
0	30 (71)	19 (86)	46 (79)
1+	7 (17)	2 (9)	10 (17)
2+	5 (12)	1 (5)	1 (2)
3+	0	0	1 (2)

a HER2 eligibility was determined by site local laboratory data.

b FISH-negative tumour was IHC 0; non-evaluable: one was IHC 0, one was IHC1+, one was IHC3+; borderline was IHC2+.

c FISH non-evaluable tumours: one was IHC1+, one was IHC2+, one was IHC3+.

d FISH borderline tumour was IHC 0.

**Table 4:** Best response by study and cohort

	**EGF100642**		**EGF104911**
	**HER2 positive (*n*=42)**	**HER2 negative (*n*=22)**	**HER2 positive (*n*=58)**
*Best response, n (%)*			
CR	0	1 (5)	1 (2)
PR	10 (24)	0	8 (14)
SD	20 (48)	3 (14)	18 (31)
PD	12 (29)	15 (68)	29 (50)
NE	0	3 (14)	2 (3)
CBR	16 (38)	1 (5)	11 (19)

Abbreviations: CR=complete response; PR=partial response; SD=stable disease; PD=progressive disease; NE=non-evaluable; CBR=clinical benefit rate (CR; PR; SD ⩾24 weeks).
